# Health complaints, social impacts, and perceived care effectiveness reported by children with obesity and their parents: a prospective observational study in the Obesity Center CGG cohort

**DOI:** 10.1007/s00431-026-07295-6

**Published:** 2026-08-01

**Authors:** Maxime M.A.M. van der Velden, Eline E.P.L. van der Walle, Patrick P.J.E Bindels, Marienke van Middelkoop, Erica E.L.T. van den Akker

**Affiliations:** 1 Department of General Practice, Erasmus MC Medical University Center, University Medical Center Rotterdam, Rotterdam, The Netherlands; 2 Obesity Center Centrum Gezond Gewicht (CGG), Erasmus Medical Center, University Medical Center Rotterdam, Rotterdam, The Netherlands; 3 Department of Pediatrics, Division of Pediatric Endocrinology, Erasmus University Medical Center, Sophia Children’s Hospital, Rotterdam, The Netherlands

**Keywords:** Obesity, Children, Lifestyle intervention, Health complaints, Social impacts

## Abstract

Obesity is a chronic relapsing disease leading to weight-related health risks for disorders such as cardiovascular diseases, type 2 diabetes, osteoarthritis, and depression. Many studies focus on these weight-related health risks. However, research on current self-reported social impacts and health complaints is limited. This study aims to investigate self-reported social impact and health complaints, received care aiming at lifestyle change, and perceived effectiveness of this care, by children with obesity and their parents. Secondarily, differences concerning sex, age, obesity-severity and frequency of received care were studied. Questionnaires were collected from children and adolescents aged 0–18 years visiting Obesity Center CGG. Data were analyzed regarding their social impacts, health complaints, received care, and perceived care effectiveness (0–10 scale). Children were categorized into sex, age, BMI, and care categories. Descriptive statistics were performed using chi-square tests and post hoc analyses to examine differences between categories. 86.8% of the patients self-reported experiencing social impact and 92.3% reported health complaints. Most frequently reported social impacts were difficulty to find fitting clothes (70.1%), problems in mobility and sports (63.7%), and bullying (41.6%); most reported health complaints were abdominal pain (38.8%), headache (34.8%), and musculoskeletal pain (34.1%). The dietician (64.1%) was most frequently reported as received care, and effectiveness scores ranged between 0 and 4 out of 10.

*Conclusions*: A substantial percentage of patients experience social impacts and health complaints affecting their well-being and quality of life. Low perceived effectiveness of interventions calls for a more innovative and comprehensive care framework incorporating these children’s needs.

**Table:** 

**What is Known:** • *Children with overweight and obesity face a wide range of adverse health consequences and social challenges.* • *However, the number and types of health issues experienced by children themselves and studying per range of age, BMI-stage or care trajectory have not been systematically investigated.*	
**What is New:** • *A high percentage of children experience social impacts (87%) and health complaints (70%) aff ecting their well-being and quality of life, and perceived eff ectiveness of lifestyle interventions is low. The main reported healthcomplaints abdominal pain, headache, musculoskeletal pain and snoring loudly were mostly observed in girls withobesity aged 12-18 years, who received three or more care types.* • *The study demonstrated that perceived treatment effectiveness by children and parents declined with increasingobesity severity.*

**Supplementary Information:**

The online version contains supplementary material available at 10.1007/s00431-026-07295-6.

## Introduction

In 2025, for the first time in history, the number of children with obesity exceeded the number of children with underweight globally [1]. Overweight and obesity have negative effects on the health and development of children [2, 3]. Conditions such as hypertension, elevated blood glucose levels, asthma, and musculoskeletal disorders are often observed in children with obesity [4]. Obesity is also associated with low self-esteem, depression, and loneliness, and children with overweight and obesity are more likely to be victims of bullying [1, 5]. These physical and psychological complications can persist into adulthood, increasing the risk of these children to develop diseases later in life such as diabetes, cardiovascular diseases and different types of cancer [6–8]. While weight-related health risks and comorbidities in children with obesity are well-studied and documented, the self-reported, current social impacts and health complaints by children with obesity remain underexplored. Moreover, insight into children’s self-reported experienced problems stratified by age and BMI stages, as well as their reported history of received care, is largely lacking. Incorporating self-reported measures is essential to capture the full scope of the burden caused by obesity in children and contributes to clinically relevant insights to the field. Given the detrimental impact of overweight and obesity during childhood on health and development, it is crucial to ensure that children with overweight and obesity receive appropriate care at the earliest possible stage. In the Netherlands, a combined lifestyle intervention (CLI) is the cornerstone of treatment; however, due to limited access, different treatment options are often sought separately such as care from a dietitian, physiotherapist, or psychologist [4, 7]. The type and intensity of the treatment provided to a child with obesity also depends upon individual factors, including the severity of the overweight, the child’s age, and needs and preferences of both the child and its family. At Obesity Center CGG (in Dutch: Centrum Gezond Gewicht) of Erasmus Medical Center (MC) and Maasstad Hospital, Rotterdam, the Netherlands, a systematic diagnostic work-up is conducted to identify the underlying causes of obesity followed by the development of a personalized treatment plan [9]. For the treatment plan, it is essential to know what type of care these children previously received and what the perceived effectiveness of that care was according to both children and their parents.

The primary aim of this study was to examine the experienced social impacts and health complaints reported by children living with overweight and obesity and their parents, the previously received care and its perceived effectiveness. Secondary, differences in these social impacts, health complaints and care across age, BMI, and care categories were explored.

## Methods

### Study design and setting

Children and adolescents 0–18 years visiting Obesity Center CGG (Dutch: *Centrum Gezond Gewicht*; English: *Centre for Healthy Weight*) were asked to participate in research. Obesity Center CGG is a Dutch multidisciplinary referral center for obesity diagnostics and care, located at Erasmus MC (academic hospital) and Maasstad Hospital (general hospital) in Rotterdam, the Netherlands. Children are referred by primary healthcare professionals (HCPs) or by pediatricians to this center for diagnostics or treatment of (severe) obesity. In this prospective, observational study, informed consent was obtained at the initial visit according to Dutch law: written informed consent was obtained from parents and children > 12 years; for children below age 12 years, oral assent was additionally obtained. The study was approved by the medical ethics committee of the Erasmus MC (MEC-2012–257).

### Study population

Prior to their first visit to the pediatric outpatient clinic at Obesity Center CGG, the children and/or their caregivers were asked to complete a questionnaire on the experienced health issues, received care, and their perceived effectiveness of this care in treating obesity. All children aged 0–18 years with an available questionnaire completed at screening were included in this study. Children with confirmed genetic obesity disorders (class 4 or 5 variants according to the ACMG criteria such as MCR4 deficiency, leptin-receptor deficiency or 16p11.2 deletion syndrome) were excluded from this study [10].

### Data collection

The structured diagnostic approach at Obesity Center CGG has been previously described in more detail [9]. For children aged 0– < 12 years, the questionnaires described above had to be completed by the caregivers. Children and adolescents aged 12–18 years were asked to complete the questionnaires themselves with caregiver assistance. The questionnaire was developed by pediatricians based on standard pediatric history taking and is included in the Dutch national pediatric practice guideline; it has been used in routine practice for ~ 20 years. Parent- and child-reported data were collected as complementary perspectives on observable and subjective symptoms. The questionnaire was administered in both paper and online formats with identical content in a comparable clinical setting. The questionnaires were completed at home, in preparation of the first outpatient clinic visit. Standardized instructions were proved to fill in the questionnaire together with the child (for children aged 0– < 12 years) or together with parents if necessary (for children aged 12–18). The questionnaires collected data regarding their age, sex, perceived age of obesity onset, perceived bodyweight, and perceived cause of the overweight/obesity. Additionally, they assessed social impacts and health issues experienced by the children (e.g., victim of bullying; physical activity limitations; asthma; abdominal pain), and the care provided to support lifestyle change received prior to the visit (e.g., dietician; general practitioner (GP); CLI) including the perceived effectiveness of that care. It should be noted that items assessing physical health complaints were introduced at a later stage of the questionnaire development. Only affirmative responses were coded as reported complaints or received care; unmarked items were interpreted as indicating the absence of the complaint or received care rather than as missing data. These reports were not based on clinical diagnoses or assessed using standardized or validated instruments but reflect self-reported experiences captured through the questionnaire. Data was collected between January 2012 and October 2025. At Maasstad Hospital, the questionnaires were completed on paper and manually entered in the database. At Erasmus MC, questionnaires were completed on paper from 2012 to 2022 and online using the program Gemstracker from 2022 onwards. As the paper and online questionnaires were identical in form and content, no assessment of administration mode effects was performed. The effectiveness scales differed between questionnaires, questionnaires collected on paper contained a 0–5 scale, while collected online contained a 0–10 scale. To enable direct comparison, scores from the 0–5 scales were multiplied by 2 to align them with the 0–10 scale with 0 representing the lowest and 10 the highest possible score.

Anthropometric measurements were performed during the first visit by trained doctor assistants. If it was not possible to perform these measurements during this visit (e.g., online intake), these measurements were performed during the second visit, when children returned for diagnostic tests. Height (cm) was determined using a wall-mounted calibrated stadiometer, and bodyweight (kg) was measured using a calibrated scale. Both measurements were rounded to the nearest decimal. Children were weighed wearing light clothing (e.g., underwear) and without shoes. The Body Mass Index (BMI) and BMI standard deviation scores (SDS) were calculated using Growth Analyzer RCT version 4.1 according to the Dutch National Growth Charts, which use the definition of obesity by Cole et al. (2000) [11]. %BMI_p95_ was calculated using the 2000 CDC Growth Charts {Ref 12,043,359}. %BMI_p95_ was used to determine the severity of overweight and obesity by classifying participants into four BMI categories: overweight (%BMI_p95_ ≥ 85 to < 100), obesity grade 1 (%BMI_p95_ ≥ 100 to < 120), obesity grade 2 (%BMI_p95_ ≥ 120 to < 140), and obesity grade 3 (%BMI_p95_ ≥ 140).

### Categorization: sex, age, BMI, and care categories

For secondary outcome analysis, children were categorized by sex (male vs. female), age (0–8; 8–12; 12–18 years), and BMI categories as described above. Children were categorized into frequency of perceived care categories based on the reported number of care actions received against their obesity before visiting CGG (0; ≤ 2; ≥ 3).

### Statistical data analysis

Descriptive statistics (mean, SD, or when the data was skewed with median, IQR) were conducted to describe the study population, reported social impacts, health complaints, received care, and perceived effectiveness.

Chi-square tests were performed to investigate differences in reported social impacts, health complaints, and received care between sex, age, BMI, and care categories. Mann–Whitney *U* tests were used to test differences between boys and girls, and Kruskal–Wallis tests were used to test differences in perceived care effectiveness between age, BMI, and care categories. Post hoc analysis with corrected residuals was conducted to investigate differences between categories.

Statistical analyses were performed using the IBM SPSS Statistics software version 15.0. Statistical significance was set at *p* < 0.05.

## Results

### Characteristics of patients

Of the 1180 children who consented to participate in research, 361 children were excluded based on the predefined exclusion criteria. A total of 819 children (69.4%) had a screening questionnaire available and were therefore included in this study. Their baseline characteristics are presented in Table 1.

**Table 1:** Characteristics of study population

Demographics	Total (*N* = 819)	0–8 years (*n* = 219)	8–12 years (*n* = 289)	12–18 years (*n* = 311)
Sex				
Boys Girls	375 (45.8%) 444 (54.2%)	94 (42.9%) 125 (57.1%)	137 (47.4%) 152 (52.6%)	144 (46.3%) 167 (53.7%)
**Age at screening (years)**	10.8 (7.8–14.3)	6.2 (4.2–7.3)	10.1 (9.1–11.1)	15.0 (13.8–16.4)
**Age of perceived onset obesity**^**a**^** (years)**	4.0 (1.5–7.0)	2.0 (0.6–4.0)	5.0 (2.0–6.5)	7.0 (3.0–10.0)
**BMI (kg/m**^**2**^**)**^**b**^	30.5 (26.1–35.7)	24.7 (22.4–27.2)	29.0 (26.3–32.5)	36.3 (32.5–40.2)
**BMI-SDS**^**b**^	3.8 (3.3–4.3)	4.1 (3.4–4.8)	3.7 (3.2–4.2)	3.8 (3.4–4.1)
**%BMIp95**^**b**^	130.5 (118.0–144.6)	131.1 (117.6–143.6)	128.2 (117.1–143.8)	132.3 (119.4–147.2)
BMI category^b^				
Overweight Obesity grade I Obesity grade II Obesity grade III	19 (2.3%) 208 (25.4%) 319 (38.9%) 270 (33.0%)	4 (1.8%) 56 (25.6%) 87 (39.7%) 72 (32.9%)	10 (3.5%) 78 (27.0%) 111 (38.4%) 89 (30.8%)	5 (1.6%) 74 (23.8%) 121 (38.9%) 109 (35.0%)
Do you think your bodyweight is too heavy?^c^				
Yes No	642 (78.4%) 143 (17.5%)	122 (55.7%) 83 (37.9%)	244 (84.4%) 31 (10.7%)	276 (88.7%) 29 (9.3%)
What do you think is the cause of your obesity?^d^				
Eating too much Physically inactive Eating too much and physically inactive Do not know, eating healthy and physically active Other	110 (13.4%) 75 (9.2%) 188 (23.0%) 218 (22.6%) 202 (24.7%)	29 (13.2%) 15 (6.8%) 28 (12.8%) 77 (35.2%) 60 (27.4%)	47 (16.3%) 30 (10.4%) 56 (19.4%) 78 (27.0%) 69 (23.9%)	34 (10.9%) 30 (9.6%) 104 (33.4%) 63 (20.3%) 73 (23.5%)
Category undertaken actions against overweight^e^				
0 care actions 2 ≤ care actions 3 ≥ care actions	118 (14.4%) 505 (61.7%) 194 (23.7%)	53 (24.2%) 130 (59.4%) 35 (16.0%)	36 (12.5%) 190 (65.7%) 62 (21.5%)	29 (9.3%) 185 (59.5%) 97 (31.2%)

Shown as frequencies (percentages); mean + SD/median + IQR. Children were classified into four BMI categories: overweight was defined as %BMI_p95_ ≥ 85 to < 100%; obesity grade I as %BMI_p95_ ≥ 100 to < 120%; grade II as %BMI_p95_ ≥ 120% to < 140% of the obesity cut-off; and grade III as %BMI_p95_ ≥ 140%

^a^There is missing data on perceived age of onset for 100 children

^b^There is missing data on BMI for 3 children

^c^There is missing data on Do you think your bodyweight is too heavy for 34 children

^d^There is missing data on What do you think is the cause of your obesity for 26 children

^e^There is missing data on Category undertaken actions against overweight for 2 children

#### Reported social impacts and health complaints

**Fig. 1:**
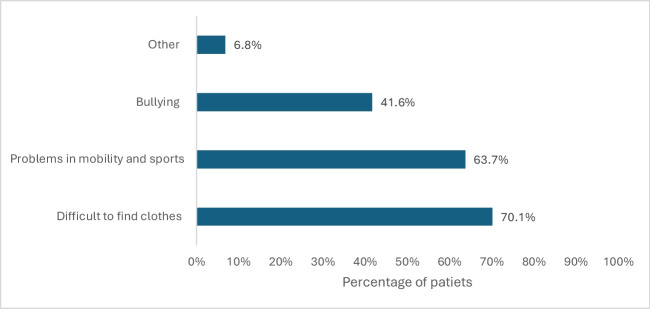
Reported social impacts by children

Of the 819 included children, 86.8% reported to experience one or more social impacts. Of the social impacts, difficulty finding fitting clothes (70.1%) was most reported, followed by problems in mobility and sports (63.7%) and bullying (41.6%) (Fig. 1). Social impacts reported as “Other” (6.8%) were often inferiority complex–related such as body-related insecurities and low self-esteem.

**Fig. 2:**
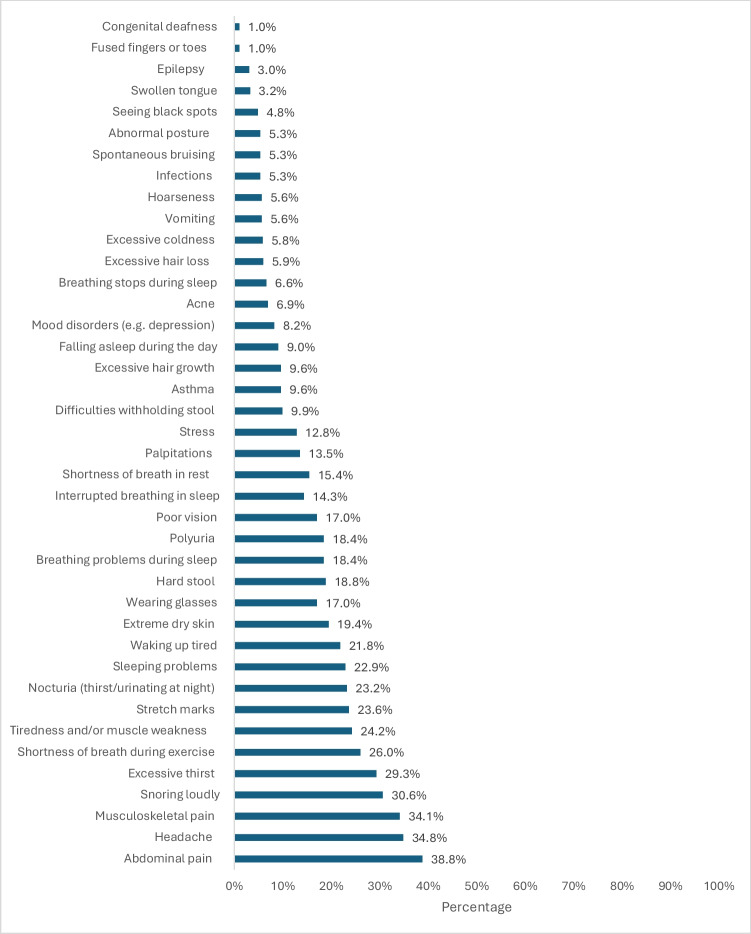
Reported health complaints by children

Of the 624 children with data on health complaints available, 92.3% reported one or more health complaints. The number of reported health complaints per child ranged from 0 to 31 (5.0; 2.0–8.0). More than a third of the children reported abdominal pain (38.8%). As shown in Fig. 2, other health complaints frequently reported were headache (34.8%), musculoskeletal pain (34.1%), snoring loudly (30.6%), excessive thirst (29.3%), and shortness of breath during exercise (26.0%).

#### Received care and effectiveness

Table 2 shows that the dietician was most frequently reported as received type of care (64.1%), followed by the physiotherapist (25.2%) and care received from the GP (17.0%). Of the children, 44.7% reported to have followed a diet themselves and only 2.9% of the children received medication (e.g., Liraglutide, Metformin, Methylphenidate) as treatment. The median reported effectiveness scores ranged between 0.5 and 4.0. For the CLI, a median effectiveness score of 4.0 (0.0–7.0) was reported. The lowest scores were reported for care received by GPs and medication (Table 2).

**Table 2:** Received care reported by children

	*N* = 819	Effectiveness (0–10 scale)
Diet	366 (44.7%)	4.0 (0.0–6.0)
General practitioner	139 (17.0%)	2.5 (0.0–5.0)
Dietician	525 (64.1%)	4.0 (0.0–6.0)
Physiotherapist	206 (25.2%)	4.0 (2.0–7.0)
Psychologist	75 (9.2%)	4.0 (1.8–6.0)
CLI	80 (9.8%)	4.0 (0.0–7.0)
Medication	24 (2.9%)	0.5 (0.0–3.8)

Shown as frequency (percentage); median (IQR). *CLI* combined lifestyle intervention

### Characteristics per categories

Children’s characteristics per category are shown in Appendix 1–12. The proportion of children perceiving their bodyweight as too heavy became higher as the age category increased but did not differ between severity of obesity grades 1–3 (Table 1). Three or more received care types were most frequently reported by children aged 12–18 years (31.2%) and BMI-Obesity III (28.6%).

#### Reported social impacts and health complaints per categories

Girls more frequently reported difficulty to find fitting clothes and bullying as social impacts (Appendix 1). Bullying was most reported by children aged 8–12 years, and problems in mobility and sports were most reported by children aged 12–18 years and with BMI-Obesity III (Appendix 4, 7). The three social impacts were most frequently reported by children who received three or more care types, compared to those who received less care types (Appendix 10).

The highest percentage for the main observed health complaints abdominal pain, headache, and musculoskeletal pain were observed in girls and children aged 12–18 years. Headache was reported significantly more often in children who received three or more care types (Appendices 2, 5, 10).

#### Received care and effectiveness per categories

All types of care were most frequently reported by children aged 12–18 years (Appendix 6). Children with Obesity grade III more often reported the care types dietician, psychologist and CLI (Appendix 9).

Care effectiveness scores showed a declining pattern with increasing weight from overweight to obesity grade III, with the highest score for care received by HCPs being the CLI and the lowest scores for GPs (Appendix 9).

## Discussion

### Main findings

This study explored the self-reported social impacts, health complaints, received care, and its perceived effectiveness in children with overweight and obesity. The majority of the children reported to experience social impact (86.8%) and health complaints (92.3%). More than half of the children experienced social impacts such as difficulty finding fitting clothes and problems in mobility and sports. The main health complaints, reported by more than 34% of the children, were abdominal and musculoskeletal pain and headache. These complaints were most frequently reported by girls, children aged 12–18 years and children who had received three or more types of care. The dietician was the main type of received care, and overall low effectiveness scores were observed with one of the lowest scores reported for care from GPs.

### Comparison with literature

In this study, 17.5% of the children did not perceive their bodyweight as too heavy. This is an interesting finding as almost all children were classified within the overweight–Obesity III range and visited a specialized center for obesity. This was mainly observed in children aged 0–8 years. It is known that especially parents’ perception regarding their child’s weight status is poor and underestimates their child’s bodyweight [12, 13]. A misperception or delayed parental recognition of their child’s overweight could affect care and its effectiveness as appropriate treatment begins with an accurate perception of overweight [14]. To provide appropriate care, parents must first accurately perceive their child as having overweight [15]. HCPs should therefore be aware of this common parental misperception, as acknowledging and addressing this bias is crucial for developing and providing effective care to this patient group.

In one study, psychosocial impacts, including difficulties in finding suitable clothes, bullying, and challenges in sports participation have been observed two to three times more often in children with overweight and obesity compared to those with a healthy bodyweight [16]. As in our study, most children (86.8%) reported to experience one or more social impacts. Seventy percent of the children reported to experience difficulties in finding clothes that fit as social impact. Finding suitable clothes can already be an issue for children with overweight from a young age, as these children often must wear clothes that are sized 2–3 years above their chronological ages [17]. The limited available sizes offered by many clothing brands often exclude children with obesity, thereby contributing to a negative body image and adverse psychological outcomes [18, 19]. Problems in mobility and sports were reported by more than half of the study population. This observation closely connects with the frequently reported health complaint musculoskeletal pain, as excessive weight during childhood increases joint loading and chronic low-grade inflammation causes muscle fatigue [11, 12]. This makes exercise for children with overweight more difficult and hinders children’s participation in sports, potentially increasing their risk of being bullied due to difficulties keeping up with peers [12, 16, 17, 20]. These findings highlight that obesity not only compromises children’s physical health but also gives rise to serious social impacts that hinder their psychosocial development and exert a substantial impact on children’s overall well-being and quality of life [16, 21].

In our study, 92.3% of the children reported one or more health complaints. For some children, this number even reached up to 31 reported complaints. The main reported health complaints included abdominal and musculoskeletal pain, headache, and many other somatic related complaints as snoring loudly, fatigue, and muscle weakness. These are serious health complaints of which evidence suggest that children with overweight and obesity experience such physical issues 3–6 times more often compared to healthy peers [22, 23]. The fact that a substantial proportion of the children reported at least one complaint underscores a considerable burden of self-reported health complaints by children with overweight and obesity. HCPs should be aware that children with overweight and obesity are not only at increased risk for future health complications but currently experience serious complaints that compromise their quality of life [24]. Integrating this knowledge into practice is crucial to work carefully with this vulnerable group of children and to tailor lifestyle-supportive interventions to the specific needs of these children [25]. This further strengthens the need for early identification and comprehensive care to prevent progressive deterioration of their current health status and to improve their quality of life.

Only 17.0% of the children indicated to have received care from their GP regarding their weight. This observation raises questions as in the Dutch healthcare system, a referral from a GP is required to get access to secondary and tertiary care like a pediatrician in settings such as Obesity Center CGG [26]. This observation may partly result from miscommunication between GPs and their patients [27]. Patients often do not perceive their GP visit as an intervention for obesity as discussions about overweight typically occur in the context of managing co-existing conditions, rather than as a primary focus [27, 28]. According to the Dutch clinical guidelines, a CLI is recommended as preferred treatment for children with overweight and obesity [26]. However, in practice, only a small proportion of the referred children had received such intervention (9.8%). This suggested that the implementation of CLI for children remains limited. Further, for almost all types of received care, low effectiveness scores were observed. These low scores were mainly reported by children with obesity grade III and those who had received two or less and three or more types of care. Although cost-effectiveness studies suggest that treatment for overweight or obesity can result in reductions in bodyweight and associated comorbidities, these improvements are generally small and report low effectiveness [29–31]. The relatively low scores are unsurprising, as they likely reflect the numerous ineffective care interventions preceding referral to CGG, an accumulation that may engender patient frustration and adversely impact health outcomes [32, 33]. Discrepancies between desired and actual health outcomes could result in disappointments and low perceived care effectiveness by patients [33–36]. This highlights the need for a clear structured care pathway available for HCPs, better communication between HCPs and their patients to manage expectations, ensure care goals, and to get a better insight into how care is perceived as effective by patients.

## Strength and limitations

While there is extensive literature available on weight-related health risks in children, studies focusing on the self-reported social impacts and health complaints experienced by children themselves are limited. As far as we know, this is the first study to investigate current social impacts, health complaints and received care by children with obesity, offering novel insights across a broad age range, BMI categories, and varying care trajectories. In this substantial sample, social impacts and health complaints were collected in a structured and standardized prospective manner. The sample is large enough for subgroup analysis. The use of self-reported questionnaires captured patients’ experiences regarding their health and care, which clinical records could miss. Though, it is important to acknowledge potential biases such as recall limitations and social desirability. Another potential limitation of this study is that several health complaints, including stress, mood disorders, and others, were not defined or assessed using standardized or validated criteria in the questionnaire, which may have resulted in varying interpretations by participants. The generalizability of these findings is uncertain. The study population included children from both secondary and tertiary specialized obesity care. Children in tertiary care often present with more severe obesity, greater comorbidity, and higher care needs than those in primary and secondary care settings. While these findings remain clinically relevant for children with obesity, they should therefore be interpreted with caution when extrapolating to children with milder overweight or to populations outside specialized obesity care. A potential limitation of this study is the use of both parent-reported data (for children < 12 years) and child-reported data (≥ 12 years). Because children younger than 12 years generally require assistance to complete questionnaires, many questionnaires were completed with parental support in this study. This represents both a strength and a limitation: while child-reported data are relatively rare, combining child self-reported and parent-reported data is an established and commonly used method in pediatric research and both are valid sources of information for health. While parents assisted with the completion of the questionnaire, the reported symptoms and health complaints were intended to reflect the child’s own experiences. Though potential differences in parent and child perceptions and reporting may have influenced the outcomes, comparisons across age groups should therefore be interpreted with caution. Another potential limitation was the use of different scales to collect perceived effectiveness of care (0–5 versus 0–10). Although these scales were harmonized, this may affect subtle differences in interpretation and sensitivity of the original scales. Further, including socio-economic status (SES) would also be highly relevant, as it may influence both experienced social impacts, health complaints and care trajectories. Future research should further explore this aspect, as it is important for healthcare professionals in tailoring care to this patient group.

## Conclusions

This study demonstrates that children with overweight and obesity visiting Obesity Center CGG frequently self-reported experiencing serious social impacts and health complaints. Besides the fact that overweight during childhood increases these children’s risk of developing health problems later in life, these children already face psychosocial and physical challenges that negatively affect their well-being and quality of life. These findings highlight the need for a more effective, comprehensive care approach, as current primary healthcare interventions focused on lifestyle change are often perceived by patients as having limited effectiveness.

## Supplementary Information

Below is the link to the electronic supplementary material.

ESM 1 (DOCX 85.6 KB)

## Data Availability

The data underlying this study are not publicly available due to privacy restrictions. Data may be made available from the corresponding author upon reasonable request.

## Abbreviations

AGMG: American College of Medical Genetics and Genomics
BMI: Body Mass Index
CLI: Combined Lifestyle Intervention
GP: General practitioner
MCR4: Melanocortin 4 receptor
MC: Medical center
SDS: Standard deviation scores
