# Supplementary material for: Health complaints, social impacts, and perceived care effectiveness reported by children with obesity and their parents: a prospective observational study in the Obesity Center CGG cohort
Source: Eur J Pediatr. 2026 Aug 1;185(8):633. doi: 10.1007/s00431-026-07295-6 (PMC13428758; doi:10.1007/s00431-026-07295-6)
Supplement: Supplementary file 1 — (DOCX 85.6 KB) [file 431_2026_7295_MOESM1_ESM.docx]

**APPENDIX**

**Appendix 1. Social impacts reported by children per sex category.**

| **What problems do you experience from having overweight or obesity?** | **Male**  **(n=375)** | **Female** **(n=444)** | **p-value** |
| --- | --- | --- | --- |
| Difficult to find clothes | 238 (63.5%) | 336 (75.7%)* | **<*0.001*** |
| Movement problems | 245 (65.3%) | 277 (62.4%) | 0.358 |
| Bullying | 143 (38.1%) | 198 (44.6%)* | **0.039** |
| Other | 20 (5.3%) | 36 (8.1%) | 0.184 |

**Shown as frequency (percentage).**
*Difficulty to find clothes and bullying were statistically significant more often reported by female patients compared to male patients.

**Appendix 2. Health complaints reported by children per sex category.**

| **What problems do you experience from having overweight or obesity?** | **Male**  **(n=287)** | **Female** **(n=337)** | **p-value** |
| --- | --- | --- | --- |
| Abdominal pain | 82 (28.6%) | 160 (47.5%)* | **<0.001** |
| Headache | 84 (29.3%) | 133 (39.5%)* | **0.006** |
| Musculoskeletal pain | 89 (31.0%) | 124 (36.8%) | 0.120 |
| Snoring loudly | 87 (30.3%) | 104 (30.9%) | 0.863 |
| Excessive thirst | 95 (33.1%) | 88 (26.1%) | 0.056 |
| Shortness of breath during exercise | 81 (28.2%) | 81 (24.0%) | 0.484 |
| Tiredness and/or muscle weakness | 66 (23.0%) | 85 (25.2%) | 0.503 |
| Stretch marks | 66 (23.0%) | 81 (24.0%) | 0.313 |
| Nocturia (thirst/urinating at night) | 62 (21.6%) | 83 (24.6%) | 0.400 |
| Sleeping problems | 60 (20.9%) | 83 (24.6%) | 0.086 |
| Wearing glasses | 57 (19.9%) | 85 (25.2%) | 0.072 |
| Waking up tired | 56 (19.5%) | 80 (23.7%)* | **0.032** |
| Extreme dry skin | 50 (17.4%) | 71 (21.1%) | 0.271 |
| Hard stool | 37 (12.9%) | 80 (23.7%)* | **<0.001** |
| Breathing problems during sleep | 56 (19.5%) | 59 (17.5%) | 0.723 |
| Polyuria | 47 (16.4%) | 68 (20.2%) | 0.220 |
| Poor vision | 44 (15.3%) | 62 (18.4%) | 0.263 |
| Interrupted breathing in sleep | 45 (15.7%) | 44 (13.1%) | 0.358 |
| Shortness of breath in rest | 48 (16.7%) | 48 (14.2%) | 0.412 |
| Palpitations | 33 (11.5%) | 51 (15.1%) | 0.180 |
| Stress | 33 (11.5%) | 47 (13.9%) | 0.168 |
| Difficulties withholding stool | 29 (10.1%) | 33 (9.8%) | 0.822 |
| Asthma | 38 (13.2%) | 22 (6.5%)* | **0.010** |
| Excessive hair growth | 16 (5.6%) | 44 (13.1%)* | **<0.001** |
| Falling asleep during the day | 21 (7.3%) | 35 (10.4%) | 0.142 |
| Mood disorders | 23 (8.0%) | 28 (8.3%) | 0.592 |
| Breathing stops during sleep | 22 (7.7%) | 19 (5.6%) | 0.382 |
| Acne | 14 (4.9%) | 29 (8.6%) | 0.058 |
| Excessive hair loss | 6 (2.1%) | 31 (9.2%)* | **<0.001** |
| Excessive coldness | 7 (2.4%) | 29 (8.6%) | **0.001** |
| Vomiting | 18 (6.3%) | 17 (5.0%) | 0.492 |
| Hoarseness | 18 (6.3%) | 17 (5.0%) | 0.502 |
| Infections | 17 (5.9%) | 16 (4.7%) | 0.678 |
| Spontaneous bruising | 16 (5.6%) | 17 (5.0%) | 0.916 |
| Abnormal posture | 18 (6.3%) | 15 (4.5%) | 0.390 |
| Seeing black spots | 11 (3.8%) | 19 (5.6%) | 0.287 |
| Swollen tongue | 12 (4.2%) | 8 (2.4%) | 0.201 |
| Epilepsy | 10 (3.5%) | 9 (2.7%) | 0.547 |
| Fused fingers or toes | 2 (0.7%) | 4 (1.2) | 0.484 |
| Congenital deafness | 2 (0.7%) | 4 (1.2%) | 0.538 |
| Fainting | 0 (0.0%) | 4 (1.2) | 0.065 |

**Shown as frequency (percentage).**
***** Abdominal pain, headache, hard stool, asthma, excessive hair growth, excessive hair loss and excessive coldness were statistically significant more reported by females compared to males.

**Appendix 3. Received care by children per sex category.**

|  | **Male** **(n=375)** | **Female** **(n=444)** | **p-value** | **Effectiveness male** | **Effectiveness female** | **p-value** |
| --- | --- | --- | --- | --- | --- | --- |
| **Diet** | 163 (43.5%) | 203 (45.7%) | 0.082 | 4.0 (0.0 – 6.0) | 3.0 (0.0-5.0) | 0.030 |
| **General practitioner** | 56 (14.9%) | 83 (18.7%) | 0.199 | 5.0 (0.0-7.0) | 1.0 (0.0-4.0) | **0.013** |
| **Dietician** | 221 (58.9%) | 304 (68.5%) | **0.008** | 4.0 (1.8 – 6.0) | 3.0 (0.0 – 5.0) | **0.021** |
| **Physiotherapist** | 98 (26.1%) | 108 (24.3%) | 0.106 | 4.0 (0.0 – 7.0) | 4.0 (2.0 – 7.0) | 0.608 |
| **Psychologist** | 38 (10.1%) | 37 (8.3%) | 0.205 | 5.0 (1.3 – 7.0) | 4.0 (1.5 – 5.3) | 0.236 |
| **CLI** | 38 (10.1%) | 42 (9.5%) | 0.224 | 3.0 (0.0 – 7.0) | 4.3 (1.0 – 7.3) | 0.560 |
| **Medication** | 13 (3.5%) | 11 (2.5%) | 0.139 | 2.0 (0.0 – 4.3) | 0.0 (0.0 – 2.5) | 0.394 |

Shown as frequency (percentage); median (IQR). CLI combined lifestyle intervention.

**Appendix 4. Social impacts reported by children per age category.**

| **What problems do you experience from having overweight or obesity?** | **0-8 years (n=219)** | **8-12 years (n=289)** | **12-18 years (n=311)** | **p-value** |
| --- | --- | --- | --- | --- |
| Difficult to find clothes that fit | 139 (63.5%) | 206 (71.3%) | 229 (73.6%) | **0.024** |
| Problems in mobility and sports | 119 (54.3%) | 188 (65.1%) | 215 (69.1%)* | **0.005** |
| Bullying | 87 (39.7%)* | 147 (50.9%) | 107 (34.4%)* | **<0.001** |
| Other | 13 (5.9%) | 12 (4.2%) | 31 (10.0%) | 0.464 |

**Shown as frequencies (percentage).**
*Posthoc analysis with adjusted residuals indicated that children aged 8-12 years reported bullying significantly more often (p<0.05); children aged 8-18 years more often reported difficulties to find clothes that fit (p<0.05); children aged 12-18 years reported problems in mobility and sports significantly more often (p<0.05).

**Appendix 5. Health complaints reported by children per age category.**

| **Type of health issue** | **0-8 years (n=182)** | **8-12 years (n=192)** | **12-18 years (n=250)** | **p-value** |
| --- | --- | --- | --- | --- |
| Abdominal pain | 55 (30.2%) | 78 (40.6%) | 109 (43.6%)* | **0.018** |
| Headache | 35 (19.2%) | 59 (30.7%) | 123 (49.2%)* | **<0.001** |
| Musculoskeletal pain | 44 (24.2%) | 71 (37.0%) | 98 (39.2%)* | **0.005** |
| Snoring loudly | 56 (30.8%) | 58 (30.2%) | 77 (30.8%) | 0.930 |
| Excessive thirst | 62 (34.1%) | 49 (25.5%) | 72 (28.8%) | 0.167 |
| Shortness of breath during exercise | 44 (24.2%) | 60 (31.3%) | 58 (23.2%) | 0.256 |
| Tiredness and/or muscle weakness | 35 (19.2%) | 48 (25.0%) | 68 (27.2%) | 0.177 |
| Stretch marks | 12 (6.6%) | 47 (24.5%) | 88 (35.2%)* | **<0.001** |
| Nocturia (thirst/urinating at night) | 57 (31.3%)* | 42 (21.9%) | 46 (18.4%) | **0.009** |
| Sleeping problems | 38 (20.9%) | 44 (22.9%) | 61 (24.4%) | 0.261 |
| Wearing glasses | 28 (15.4%) | 34 (17.7%) | 80 (32.0%)* | **<0.001** |
| Waking up tired | 33 (18.1%) | 35 (18.2%) | 68 (27.2%)* | **0.001** |
| Extreme dry skin | 44 (24.2%) | 33 (17.2%) | 44 (17.6%) | 0.148 |
| Hard stool | 25 (13.7%) | 41 (21.4%) | 51 (20.4%) | 0.147 |
| Breathing problems during sleep | 36 (19.8%) | 37 (19.3%) | 42 (16.8%) | 0.606 |
| Polyuria | 45 (24.7%) | 36 (18.8%) | 34 (13.6%) | **0.010** |
| Poor vision | 21 (11.5%) | 27 (14.1%) | 58 (23.2%)* | **0.002** |
| Interrupted breathing in sleep | 29 (15.9%) | 29 (15.1%) | 31 (12.4%) | 0.653 |
| Palpitations | 17 (9.3%) | 20 (10.4%) | 47 (18.8%)* | **0.007** |
| Stress | 17 (9.3%) | 19 (9.9%) | 44 (17.6%)* | **0.001** |
| Difficulties withholding stool | 21 (11.5%) | 25 (13.0%) | 16 (6.4%) | 0.054 |
| Shortness of breath during rest | 24 (13.2%) | 25 (13.0%) | 47 (18.8%) | 0.157 |
| Asthma | 20 (11.0%) | 17 (8.9%) | 23 (9.2%) | 0.852 |
| Excessive hair growth | 12 (6.6%) | 17 (8.9%) | 31 (12.4%) | 0.060 |
| Falling asleep during the day | 16 (8.8%) | 8 (4.2%) | 32 (12.8%)* | **0.007** |
| Mood disorders | 13 (7.1%) | 8 (4.2%) | 30 (12.0%)* | **0.002** |
| Acne | 5 (2.7%) | 6 (3.1%) | 32 (12.8%)* | **<0.001** |
| Breathing stops during sleep | 10 (5.5%) | 15 (7.8%) | 16 (6.4%) | 0.339 |
| Excessive hair loss | 7 (3.8%) | 5 (2.6%) | 25 (10.0%)* | **0.002** |
| Excessive coldness | 8 (4.4%) | 6 (3.1%) | 22 (8.8%) | **0.029** |
| Vomiting | 16 (8.8%) | 8 (4.2%) | 11 (4.4%) | 0.082 |
| Hoarseness | 11 (6.0%) | 8 (4.2%) | 16 (6.4%) | 0.579 |
| Infections | 9 (4.9%) | 11 (5.7%) | 13 (5.2%) | 0.985 |
| Spontaneous bruising | 14 (7.7%) | 10 (5.2%) | 9 (3.6%) | 0.166 |
| Abnormal posture | 5 (2.7%) | 11 (5.7%) | 17 (6.8%) | 0.131 |
| Seeing black spots | 3 (1.6%) | 9 (4.7%) | 18 (7.2%) | 0.028 |
| Swollen tongue | 4 (2.2%) | 4 (2.1%) | 12 (4.8%) | 0.184 |
| Epilepsy | 5 (2.7%) | 4 (2.1%) | 10 (4.0%) | 0.494 |
| Fused fingers or toes | 1 (0.5%) | 2 (1.0%) | 3 (1.2%) | 0.756 |
| Congenital deafness | 1 (0.5%) | 1 (0.5%) | 4 (1.6%) | 0.412 |
| Fainting | 0 (0.0%) | 0 (0.0%) | 4 (1.6%) | 0.050 |

Shown as frequency (percentage).
*Post-hoc analysis with corrected residuals indicates that age groups 0-8 years reported nocturia and polyuria significantly more often (p<0.05); abdominal pain, musculoskeletal pain, stretch marks, headache, palpitations, asthma, acne, depression, stress, mood disorders, excessive hair loss, excessive coldness, falling asleep during the day, waking up tired, poor vision, seeing black spots and wearing glasses were significantly more frequently reported by age group 12-18 years.

**Appendix 6. Received care and perceived effectiveness reported by children per age category.**

|  | **0-8 years (n=219)** | **8-12 years (n=289)** | **12-18 years (n=311)** | **p-value** | **Effectiveness 0-8 years (0-10 scale)** | **Effectiveness 8-12 years (0-10 scale)** | **Effectiveness 12-18 years (0-10 scale)** | **p-value** |
| --- | --- | --- | --- | --- | --- | --- | --- | --- |
| Diet | 83 (37.9%) | 133 (46.0%) | 150 (48.2%) | **0.049** | 2.0 (0.0 – 5.0) | 4.0 (0.0 – 6.0) | 4.0 (1.0 – 6.0) | 0.361 |
| General practitioner | 26 (11.9%) | 54 (18.7%) | 59 (19.0%) | 0.053 | 2.5 (0.0 – 7.3) | 3.0 (0.0 – 6.0) | 1.5 (0.0 – 4.3) | 0.492 |
| Dietician | 114 (52.1%) | 183 (63.3%) | 228 (73.3%)* | **<0.001** | 2.0 (0.0 – 6.0) | 4.0 (0.0 – 6.0) | 4.0 (0.0 – 5.0) | 0.421 |
| Physiotherapist | 56 (25.6%) | 67 (23.2%) | 83 (26.7%) | 0.223 | 4.0 (0.8 – 7.0) | 4.0 (2.0 – 7.0) | 5.0 (2.0 – 7.0) | 0.975 |
| Psychologist | 11 (5.0%) | 20 (6.9%) | 44 (14.1%)* | **<0.001** | 0.0 (0.0 – 6.0) | 3.5 (0.0 – 5.5) | 4.0 (2.5 –6.5) | 0.258 |
| CLI | 9 (4.1%) | 24 (8.3%) | 47 (15.1%)* | **<0.001** | 2.5 (0.0 –7.3) | 2.0 (0.0 –6.0) | 4.0 (1.3 – 7.0) | 0.679 |
| Medication | 2 (0.9%) | 6 (2.1%) | 16 (5.1%)* | **0.004** | / | 0.0 (0.0 – 0.0) | 2.0 (0.3 – 4.8) | 0.093 |

Shown as frequencies (percentages); median + IQR. CLI combined lifestyle intervention.
*Post-hoc analysis with corrected residuals indicates that care received by the dietician, psychologist, CLI and medication were statistically significant more frequently reported by age group 12-18 years.

**Appendix 7. Social impacts reported by children per BMI category.**

| **What problems do you experience from having overweight or obesity?** |  | **Overweight** **(n=19)** | **Obesity I** **(n=208)** | **Obesity II** **(n=319)** | **Obesity III** **(n=270)** | **p-value** |  |
| --- | --- | --- | --- | --- | --- | --- | --- |
| Difficult to find clothes |  | 11 (57.9%) | 142 (68.3%) | 221 (69.3%) | 197 (73.0%) | 0.575 |  |
| Problems in mobility and sports |  | 12 (63.2%) | 121 (58.2%) | 205 (64.3%) | 181 (67.0%) | 0.157 |  |
| Bullying |  | 8 (42.1%) | 75 (36.1%) | 131 (41.1%) | 125 (46.3%) | 0.095 |  |
| Other |  | 0 (0.0%) | 14 (6.7%) | 26 (8.2%) | 16 (5.9%) | **0.046** |  |

Shown as frequency (percentage).
*Posthoc analysis with adjusted residuals indicated that children with Obesity II reported other social impact significantly more often (p<0.05);

**Appendix 8. Health complaints reported by children per BMI category.**

| **Type of health issue** | **Overweight** **(n=11)** | **Obesity I** **(n=152)** | **Obesity II** **(n=253)** | **Obesity III** **(n=208)** | **p-value** |
| --- | --- | --- | --- | --- | --- |
| Abdominal pain | 4 (36.4%) | 55 (36.2%) | 98 (38.7%) | 85 (40.9%) | 0.760 |
| Headache | 4 (36.4%) | 55 (36.2%) | 85 (33.6%) | 73 (35.1%) | 0.928 |
| Musculoskeletal pain | 3 (27.3%) | 58 (38.2%) | 85 (33.6%) | 67 (32.2%) | 0.631 |
| Snoring loudly | 3 (27.3%) | 33 (21.7%) | 70 (27.7%) | 85 (40.9%)* | **<0.001** |
| Excessive thirst | 3 (27.3%) | 39 (25.7%) | 71 (28.1%) | 70 (33.7%) | 0.363 |
| Shortness of breath during exercise | 1 (9.1%) | 31 (20.4%) | 63 (24.9%) | 67 (32.2%) | 0.056 |
| Tiredness and/or muscle weakness | 3 (27.3%) | 43 (28.3%) | 54 (21.3%) | 51 (24.5%) | 0.475 |
| Stretch marks | 0 (0.0%) | 30 (19.7%) | 53 (20.9%) | 64 (30.8%)* | **0.020** |
| Nocturia (thirst/urinating at night) | 3 (27.3%) | 37 (24.3%) | 51 (20.2%) | 54 (26.0%) | 0.460 |
| Sleeping problems | 3 (27.3%) | 36 (23.7%) | 51 (20.2%) | 53 (25.5%) | 0.364 |
| Wearing glasses | 4 (36.4%) | 36 (23.7%) | 51 (20.2%) | 51 (24.5%) | 0.433 |
| Waking up tired | 1 (9.1%) | 27 (17.8%) | 58 (22.9%) | 50 (24.0%) | 0.596 |
| Extreme dry skin | 0 (0.0%) | 34 (22.4%) | 45 (17.8%) | 42 (20.2%) | 0.286 |
| Hard stool | 3 (27.3%) | 29 (19.1%) | 55 (21.7%) | 30 (14.4%) | 0.216 |
| Breathing problems during sleep | 2 (18.2%) | 20 (13.2%) | 36 (14.2%) | 57 (27.4%)* | **0.009** |
| Polyuria | 3 (27.3%) | 41 (27.0%)* | 37 (14.6%) | 34 (16.3%) | **0.010** |
| Poor vision | 2 (18.2%) | 28 (18.4%) | 38 (15.0%) | 38 (18.3%) | 0.730 |
| Shortness of breath at rest | 0 (0.0%) | 22 (14.5%) | 33 (13.0%) | 41 (19.7%) | 0.094 |
| Interrupted breathing in sleep | 0 (0.0%) | 14 (9.2%) | 30 (11.9%) | 45 (21.6%)* | **<0.001** |
| Palpitations | 1 (9.1%) | 15 (9.9%) | 37 (14.6%) | 31 (14.9%) | 0.463 |
| Stress | 1 (9.1%) | 22 (14.5%) | 32 (12.6%) | 25 (12.0%) | 0.788 |
| Difficulties withholding stool | 2 (18.2%) | 11 (7.2%) | 18 (7.1%) | 31 (14.9%)* | **0.008** |
| Asthma | 1 (9.1%) | 14 (9.2%) | 23 (9.1%) | 22 (10.6%) | 0.976 |
| Excessive hair growth | 0 (0.0%) | 15 (9.9%) | 23 (9.1%) | 22 (10.6%) | 0.870 |
| Falling asleep during the day | 0 (0.0%) | 10 (6.6%) | 18 (7.1%) | 28 (13.5%) | 0.060 |
| Mood disorders | 1 (9.1%) | 9 (5.9%)* | 20 (7.9%) | 21 (10.1%) | 0.501 |
| Acne | 0 (0.0%) | 9 (5.9%) | 17 (6.7%) | 17 (8.2%) | 0.832 |
| Breathing stops during sleep | 0 (0.0%) | 5 (3.3%) | 10 (4.0%) | 26 (12.5%)* | **0.001** |
| Excessive hair loss | 0 (0.0%) | 8 (5.3%) | 14 (5.5%) | 15 (7.2%) | 0.689 |
| Excessive coldness | 2 (18.2%) | 10 (6.6%) | 9 (3.6%) | 15 (7.2%) | 0.075 |
| Hoarseness | 1 (9.1%) | 10 (6.6%) | 12 (4.7%) | 12 (4.7%) | 0.794 |
| Vomiting | 1 (9.1%) | 6 (3.9%) | 12 (4.7%) | 16 (7.7%) | 0.349 |
| Infections | 1 (9.1%) | 8 (5.3%) | 17 (6.7%) | 7 (3.4%) | 0.131 |
| Abnormal posture | 1 (9.1%) | 5 (3.3%) | 14 (5.5%) | 13 (6.3%) | 0.318 |
| Spontaneous bruising | 0 (0.0%) | 5 (3.3%) | 14 (5.5%) | 14 (6.7%) | 0.492 |
| Seeing black spots | 0 (0.0%) | 8 (5.3%) | 13 (5.1%) | 9 (4.3%) | 0.864 |
| Swollen tongue | 0 (0.0%) | 2 (1.3%) | 7 (2.8%) | 10 (4.8%) | 0.169 |
| Epilepsy | 0 (0.0%) | 5 (3.3%) | 7 (2.8%) | 7 (3.4%) | 0.923 |
| Fused fingers or toes | 0 (0.0%) | 2 (1.3%) | 3 (1.2%) | 1 (0.5%) | 0.784 |
| Congenital deafness | 0 (0.0%) | 2 (1.3%) | 2 (0.8%) | 2 (1.0%) | 0.948 |
| Fainting | 0 (0.0%) | 3 (2.0%) | 0 (0.0%) | 1 (0.5%) | 0.114 |

**Shown as frequency (percentage).**
*Posthoc analysis with adjusted residuals indicated thatchildren with Obesity I reported polyuria significantly more often (p<0.05); children with Obesity III reported stretch marks, snoring loudly, breathing stops during sleep, interrupted breathing in sleep and problems withholding stool significantly more often (p<0.05).

**Appendix 9. Received care and perceived effectiveness reported by children per BMI category.**

|  | **Overweight** **(n=19)** | **Obesity I** **(n=208)** | **Obesity II** **(n=319)** | **Obesity III** **(n=270)** | **p-value** | **Effectiveness overweight (0-10 scale)** | **Effectiveness obesity I (0-10 scale)** | **Effectiveness obesity II (0-10 scale)** | **Effectiveness obesity III** | **p-value** |
| --- | --- | --- | --- | --- | --- | --- | --- | --- | --- | --- |
| **Diet** | 10  (52.6%) | 86 (41.3%) | 145 (45.5%) | 122 (45.2%) | 0.923 | 6.0 (0.0 – 5.0) | 4.0 (2.0 – 6.0) | 4.0 (0.0 – 6.0) | 3.0 (0.0 – 5.0) | 0.220 |
| **General practitioner** | 4 (21.1%) | 30 (14.4%) | 49 (15.4%) | 56 (20.7%) | 0.347 | . | 0.0 (0.0 – 3.8) | 0.0 (0.0 – 5.0) | 4.0 (0.0 – 6.0) | 0.541 |
| **Dietician** | 8 (42.1%) | 120 (57.7%) | 198 (62.1%) | 197 (73.0%)* | **0.013** | 0.0 (0.0 – 6.0) | 4.0 (0.0 – 6.0) | 4.0 (0.0 – 6.0) | 4.0 (0.0 – 5.3) | 0.579 |
| **Physiotherapist** | 1 (5.3%) | 49 (23.6%) | 78 (24.5%) | 78 (28.9%) | 0.335 | . | 5.0 (2.0 – 7.0) | 4.0 (2.0 – 7.0) | 4.0 (20 – 7.0) | 0.240 |
| **Psychologist** | 0 (0.0%) | 10 (4.8%) | 29 (9.1%) | 36 (13.3%)* | **0.023** | . | 2.0 (1.0 – 5.9) | 5.0 (3.0 –7.0) | 4.0 (0.3 – 4.7) | 0.268 |
| **CLI** | 1 (5.3%) | 16 (7.7%) | 27 (8.5%) | 36 (13.3%)* | **0.044** | . | 4.0 (2.0 –6.5) | 2.0 (0.0 – 4.7) | 6.0 (1.3 – 8.0) | 0.156 |
| **Medication** | 0 (0.0%) | 9 (4.3%) | 7 (2.2%) | 8 (3.0%) | 0.395 | . | 1.0 (0.0 – 4.0) | 0.0 (0.0 – 3.0) | 1.0 (0.0 – .) | 0.421 |

**Shown as frequency (Percentage); median (IQR).** CLI combined lifestyle intervention.
* Posthoc analysis with adjusted residuals indicated children that with Obesity-III significantly more often reported having received the care types dietician, psychologist and CLI (p<0.05).

**Appendix 10. Social impacts reported by children per received care category.**

| **What problems do you experience from having overweight or obesity?** | **0 care actions (n=118)** | **2≤ care actions**  **(n=505)** | **3≥ care actions (n=194)** | **p-value** |
| --- | --- | --- | --- | --- |
| Difficult to find clothes | 67 (56.8%) | 352 (69.7%) | 154 (79.4%)* | **<0.001** |
| Problems in mobility and sports | 58 (49.2%) | 316 (62.6%) | 147 (75.8%)* | **<0.001** |
| Bullying | 42 (35.6%) | 202 (40.0%) | 96 (49.5%)* | **0.034** |
| Other | 0 (0.0%) | 32 (6.3%) | 24 (12.4%) | 0.280 |

Shown as frequency (percentage).
*Posthoc analysis with adjusted residuals indicated that children who receive three or more care actions reported difficulty to find clothes, problems in mobility and sports and bullying statistically significant more often (p<0.05).

**Appendix 11. Health complaints reported by children per received care category.**

| **What problems do you experience from having overweight or obesity?** | **0 care actions (n=92)** | **2≤ care actions**  **(n=381)** | **3≥ care actions (n=149)** | **p-value** |
| --- | --- | --- | --- | --- |
| Abdominal pain | 32 (34.8%) | 146 (38.3%) | 64 (43.0%) | 0.483 |
| Headache | 20 (21.7%) | 128 (33.6%) | 69 (46.3%)* | **<0.001** |
| Musculoskeletal pain | 28 (30.4%) | 125 (32.8%) | 60 (40.3%) | 0.230 |
| Snoring loudly | 26 (28.3%) | 111 (29.1%) | 53 (35.6%) | 0.341 |
| Excessive thirst | 23 (25.0%) | 114 (29.9%) | 46 (30.9%) | 0.621 |
| Shortness of breath during exercise | 17 (18.5%) | 98 (25.7%) | 47 (31.5%) | 0.557 |
| Stretch marks | 10 (10.9%) | 80 (21.0%) | 57 (38.3%)* | **<0.001** |
| Tiredness and/or muscle weakness | 17 (18.5%) | 89 (23.4%) | 45 (30.2%) | 0.119 |
| Nocturia (thirst/urinating at night) | 24 (26.1%) | 84 (22.0%) | 37 (24.8%) | 0.654 |
| Sleeping problems | 14 (15.2%) | 84 (22.0%) | 45 (30.2%) | 0.318 |
| Wearing glasses | 18 (19.6%) | 88 (23.1%) | 36 (24.4%) | 0.661 |
| Waking up tired | 11 (12.0%) | 83 (21.8%) | 42 (28.2%) | 0.217 |
| Breathing problems during sleep | 15 (16.3%) | 77 (20.2%) | 23 (15.4%) | 0.129 |
| Extreme dry skin | 12 (13.0%) | 75 (19.7%) | 34 (22.8%) | 0.198 |
| Hard stool | 20 (21.7%) | 69 (18.1%) | 28 (18.8%) | 0.694 |
| Polyuria | 14 (15.2%) | 69 (18.1%) | 31 (20.8%) | 0.579 |
| Poor vision | 13 (14.1%) | 62 (16.3%) | 31 (20.8%) | 0.422 |
| Shortness of breath at rest | 9 (9.8%) | 61 (16.0%) | 26 (17.4%) | 0.258 |
| Interrupted breathing in sleep | 12 (13.0%) | 55 (14.4%) | 22 (14.8%) | 0.936 |
| Palpitations | 6 (6.5%) | 52 (13.6%) | 26 (17.4%) | 0.061 |
| Stress | 8 (8.7%) | 42 (11.0%) | 30 (20.1%)* | **0.026** |
| Difficulties withholding stool | 7 (7.6%) | 42 (11.0%) | 13 (8.7%) | 0.558 |
| Asthma | 6 (6.5%) | 40 (10.5%) | 14 (9.4%) | 0.474 |
| Excessive hair growth | 6 (6.5%) | 37 (9.7%) | 17 (11.4%) | 0.919 |
| Falling asleep during the day | 4 (4.3%) | 37 (9.7%) | 15 (10.1%) | 0.787 |
| Mood disorders | 2 (2.2) | 25 (6.6%) | 24 (16.1%)* | **<0.001** |
| Acne | 2 (2.2%) | 27 (7.1%) | 14 (9.4%) | 0.524 |
| Breathing stops during sleep | 5 (5.4%) | 25 (6.6%) | 11 (7.4%) | **0.019** |
| Excessive hair loss | 2 (2.2%) | 27 (7.1%) | 8 (5.4%) | 0.236 |
| Excessive coldness | 5 (5.4%) | 20 (5.2%) | 11 (7.4%) | 0.634 |
| Vomiting | 9 (9.8%)* | 24 (6.3%) | 2 (1.3%) | **0.012** |
| Hoarseness | 3 (3.3%) | 21 (5.5%) | 11 (7.4%) | 0.404 |
| Infections | 2 (2.2%) | 19 (5.0%) | 12 (8.1%) | 0.336 |
| Spontaneous bruising | 4 (4.3%) | 18 (4.7%) | 11 (7.4%) | 0.579 |
| Abnormal posture | 2 (2.2%) | 19 (5.0%) | 12 (8.1%) | 0.280 |
| Seeing black spots | 2 (2.2%) | 18 (4.7%) | 10 (6.7%) | 0.273 |
| Swollen tongue | 2 (2.2%) | 14 (3.7%) | 4 (2.7%) | 0.697 |
| Fused fingers or toes | 1 (1.1%) | 4 (1.0%) | 1 (0.7%) | 0.832 |
| Congenital deafness | 0 (0.0%) | 4 (1.0%) | 2 (1.3%) | 0.567 |
| Epilepsy | 1 (1.1%) | 15 (3.9%) | 3 (2.0%) | 0.257 |
| Fainting | 1 (1.1%) | 2 (0.5%) | 1 (0.7%) | 0.829 |

**Shown as frequencies (percentage).**
*Posthoc analysis with adjusted residuals indicated that children who receive zero care actions reported vomiting significantly more often (p<0.05); children who received three or more care actions reported stretch marks, headache, stressand mood disorders significantly more often (p<0.05).

**Appendix 12. Received care and perceived effectiveness reported by children per care group.**

|  | **2≤ care actions**  **(n=505)** | **3≥ care actions (n=194)** | **p-value** | **Effectiveness two or less (0-10 scale)** | **Effectiveness three or more (0-10 scale)** | **p-value** |
| --- | --- | --- | --- | --- | --- | --- |
| **Diet** | 207 (41.0%) | 159 (82.0%)* | **<0.001** | 4.0 (0.0–6.0) | 4.0 (0.0–6.0) | 0.751 |
| **General practitioner** | 39 (7.7%) | 100 (51.5%)* | **<0.001** | 2.0 (0.0–5.0) | 3.0 (0.0–5.3) | 0.678 |
| **Dietician** | 337 (66.7%) | 188 (96.9%)* | **<0.001** | 4.0 (0.0–6.0) | 4.0 (0.0–6.0) | 0.983 |
| **Physiotherapist** | 77 (15.2%) | 129 (66.5%)* | **<0.001** | 5.0 (2.0–6.0) | 4.0 (2.0–7.0) | 0.380 |
| **Psychologist** | 14 (2.8%) | 61 (31.4%)* | **<0.001** | 4.0 (2.3–7.0) | 4.0 (2.0 – 6.0) | 0.866 |
| **CLI** | 31 (6.1%) | 49 (25.3%)* | **<0.001** | 4.5 (0.0–8.0) | 4.0 (1.0–6.0) | 0.514 |
| **Medication** | 10 (2.0%) | 14 (7.2%)* | **<0.001** | 2.5 (0.0-6.0) | 1.0 (0.0-2.0) | 0.551 |

**Shown as frequencies (percentages); median + IQR.** CLI combined lifestyle intervention.
*Posthoc analysis with adjusted residuals indicated that children who receive three or more care actions reported all care types significantly more (p<0.05).
